# In‐hospital mortality in dogs with protein‐losing enteropathy and associated risk factors

**DOI:** 10.1111/jvim.17123

**Published:** 2024-05-31

**Authors:** Connor Hawes, Aarti Kathrani

**Affiliations:** ^1^ Royal Veterinary College University of London London UK

**Keywords:** canine, C‐reactive, diarrhea, gastrointestinal, pneumonia, pug

## Abstract

**Background:**

Risk factors associated with negative outcomes in dogs with protein‐losing enteropathy (PLE) are well documented. However, mortality before hospital discharge and associated risk factors are not well described.

**Hypothesis/Objectives:**

Report the percentage of dogs with PLE that do not survive to hospital discharge and identify associated risk factors.

**Animals:**

One‐hundred and seven dogs presented to a referral hospital and diagnosed with PLE caused by inflammatory enteritis, intestinal lymphangiectasia or both.

**Methods:**

Retrospective cross‐sectional study assessing hospital records. Data on in‐hospital mortality and cause were assessed, and presenting signs, treatments prescribed, neutrophil count, lymphocyte count, serum albumin, globulin, and C‐reactive protein (CRP) concentrations, and histopathologic findings were compared between survivors and non‐survivors.

**Results:**

In‐hospital mortality was 21.5% with the most common causes including financial limitations, failure to improve and aspiration pneumonia. Factors associated with mortality during hospitalization included longer duration of hospitalization (*P* = .04), longer duration of clinical signs (*P* = .02) and an increase in serum CRP concentration after 1–3 days of in‐hospital treatment (*P* = .02). Higher mortality was identified in Pugs (odds ratio [OR], 4.93; 95% confidence interval [CI], 1.41–17.2; *P* = .01) and was a result of presumptive aspiration pneumonia in 5/6 of these dogs.

**Conclusions and Clinical Importance:**

Protein‐losing enteropathy in dogs has substantial mortality during hospitalization. Monitoring for improvement in CRP concentration after treatment during hospitalization may help predict survival to discharge. Pugs have increased in‐hospital mortality because of aspiration pneumonia; measures to prevent, recognize, and promptly treat this complication may improve outcomes in this breed.

**Abbreviations**
CRP: C‐reactive protein
NLR: neutrophil‐to‐lymphocyte ratio
PLE: protein‐losing enteropathy

## 1 INTRODUCTION

Protein‐losing enteropathy (PLE) is a syndrome in which the rate of loss of serum protein across the intestinal wall exceeds the rate of protein synthesis, resulting in hypoproteinemia.^1^ It is a debilitating condition, resulting in disease‐associated death in 50% of affected dogs.^1^, ^2^ Several factors associated with negative outcome in dogs with PLE have been identified, including severity of hypoalbuminemia, hypocobalaminemia, hypovitaminosis D, canine chronic enteropathy clinical activity index (CCECAI), body weight, increased blood urea nitrogen concentration, small intestinal dilatation, epaxial muscle loss, coat condition, and C‐reactive protein (CRP) concentration.^3^, ^4^, ^5^, ^6^, ^7^, ^8^, ^9^, ^10^

Although the rate of mortality of dogs with PLE has been well documented, the cause of death often is not identified. Complications associated with PLE that may contribute to mortality include hypercoagulability, which has been documented in 45% of dogs with chronic enteropathy^11^ and was suspected to be the cause of mortality in 10% of Yorkshire terriers with PLE,^12^ effusions, immunodeficiency,^13^ and hypocalcemia.^14^ Failure to respond to treatment also may result in the decision to euthanize affected dogs. In addition, complications such as hypercoagulability and immunosuppression may be exacerbated by treatments such as prednisolone^15^ and by obtaining intestinal biopsy specimens, which are often required to make a definitive diagnosis. Despite several studies evaluating prognostic factors, few studies have explored causes of in‐hospital mortality in dogs with PLE. Such information will help identify dogs at risk of in‐hospital mortality and causative factors, so that closer monitoring and treatment goals can be established during hospitalization.

Our hypothesis was that PLE was associated with substantial mortality before hospital discharge, and that factors associated with severity of disease at the time of presentation might predict survival. Our aims were to determine the percentage of dogs with PLE caused by inflammatory enteritis, lymphangiectasia or both that did not survive to hospital discharge, document causes of mortality, and identify any associated risk factors.

## 2 MATERIALS AND METHODS

### 2.1 Case selection

The medical records at the Queen Mother Hospital for Animals, Royal Veterinary College were retrospectively searched between July 2010 and July 2023 for dogs diagnosed with PLE caused by inflammatory enteritis, lymphangiectasia or both. Inclusion criteria comprised clinical signs consistent with PLE, hypoalbuminemia (<26.3 g/L) at presentation and histopathology of intestinal biopsy specimens obtained either surgically or endoscopically that documented changes consistent with inflammatory enteritis, lymphangiectasia or both. Cases were excluded if intestinal neoplasia had been identified on histopathology, if histopathology had not been performed or if initial CBC, serum biochemistry, urinalysis, and imaging results had failed to exclude hypoadrenocorticism or hepatic and renal causes of hypoalbuminemia. In total, 107 dogs met the criteria and were included in the study.

### 2.2 Data collection

Signalment, body weight (kg), presenting clinical signs, duration of clinical signs (months), and treatment within 2 months before presentation were recorded. Treatment before presentation was categorized as no antibiotics or immunosuppressants, immunosuppressants alone, antibiotics alone, or a combination of antibiotics and immunosuppressants. Details regarding dietary management before presentation was not included, because of difficulty in reliably obtaining this information from previous medical records.

Hospital records and results of diagnostic investigations were reviewed. The information recorded consisted of body condition score (BCS), neutrophil count, lymphocyte count, and neutrophil‐to‐lymphocyte ratio (NLR), and serum albumin, globulin, and CRP concentrations. Where serum biochemistry analysis had been repeated after beginning treatment and during hospitalization, CRP concentrations from these results also were recorded. Duodenal, colonic, and ileal histopathology was reviewed, and the identified inflammatory cells were recorded (lymphocytic, plasmacytic, neutrophilic, eosinophilic, and histiocytic), as well as the presence of lymphangiectasia, crypt abscess, and lacteal dilatation where available. Where the laboratory histopathology report was not available for review, the histopathologic diagnosis that was recorded in the discharge report was used.

Treatment during hospitalization and duration of hospitalization (days) as determined from the date of admission to the date of discharge or death also were recorded. In‐hospital treatments were categorized as the following or a combination of these: diet, antibiotics, glucocorticoids, and second immunosuppressive agent. Second agents were considered to be any immunosuppressant drug that was not a glucocorticoid. Diets were categorized as the following: commercial therapeutic gastrointestinal, commercial therapeutic hydrolyzed, commercial therapeutic limited ingredient novel protein or other. Cause of death was defined as that recorded on the hospital discharge report. When the dog had been euthanized, the cause of death was recorded as the reason for euthanasia or reason for deterioration. Because we exclusively used retrospective case data from hospital records, ethical approval was not required as determined by our hospital's ethical review board; Clinical Research Ethical Review Board at the Royal Veterinary College.

### 2.3 Statistical analysis

A commercially available computer software package (IBM SPSS Statistics Version 29) was utilized for all statistical analyses. Histograms and the Shapiro‐Wilk test were performed to assess for normality of continuous variables. Results were reported as mean (SD) if normally distributed and as median (range) if not normally distributed.

To compare continuous data (age, body weight, BCS, duration of hospitalization, duration of clinical signs before admission, prednisolone dose, neutrophil count, lymphocyte count, NLR, serum albumin, globulin, and CRP concentrations) between dogs that did and did not survive to hospital discharge, independent sample *t*‐test and Mann‐Whitney *U* test were utilized for parametric and non‐parametric data, respectively. Pearson's Chi‐squared test was used to compare categorical data (breed, signs, treatment before admission, treatment during hospitalization, diet consumed during hospitalization, and histopathologic diagnosis) between survivors and non‐survivors. Odds ratios (OR) were reported along with 95% confidence intervals (CI) where appropriate. A type I error rate of .05 was utilized for all statistical analyses.

## 3 RESULTS

### 3.1 Signalment

One‐hundred and seven dogs met the inclusion criteria. The mean age at presentation was 7.1 +/−3.1 years. Fifteen were male intact, 51 male neutered, 5 female intact, and 36 female neutered. There were 37 different breeds recorded, the most common being: Staffordshire Bull Terrier (14), Pug (12), cross breed (12), Cavalier King Charles Spaniel (6), Bichon Frise (5), Miniature Schnauzer (4), German Shepherd (4), Bulldog (4), Dachshund (3), Cocker Spaniel (3), Jack Russell Terrier (3), Labrador Retriever (3), Rottweiler (3), Border Collie (2), Boxer (2), Brussels Griffon (2), Dogue De Bordeaux (2), Golden Retriever (2), Hungarian Vizsla (2), Whippet (2), and Yorkshire Terrier (2). There also were 1 of each of the following breeds: Airedale Terrier, Basset Fauve De Bretagne, Beagle, Chihuahua, Cockapoo, English Mastiff, English Springer Spaniel, Fox Terrier, German Shorthair Pointer, Leonberger, Neapolitan Mastiff, Shetland Sheepdog, Shiba Inu, Tibetan Terrier, and Weimaraner.

### 3.2 Clinical signs and previous treatments

The median duration of clinical signs was 1.5 (range, 0.1–42) months. The most commonly reported clinical signs were: diarrhea in 93/107 (86.9%), weight loss in 36/107 (33.6%), vomiting in 34/107 (31.8%), and decreased appetite in 27/107 (25.2%) dogs. Information on prior treatment with antibiotics or immunosuppressants within 2 months of presentation was available for 99/107 dogs. Twenty‐five dogs (25%) had received no antibiotics or immunosuppressants, 49 (49%) had received antibiotics, 6 (6%) had received immunosuppressants, all of which were glucocorticoids, and 19 (19%) had received both antibiotics and immunosuppressants, all of which were glucocorticoids.

### 3.3 Diagnostic investigations

Intestinal histopathology had been performed in all dogs. One‐hundred and three (96%) intestinal biopsy specimens had been obtained endoscopically and 4 (3.7%) were obtained surgically. Of the 103 dogs in which endoscopic biopsy specimens were obtained, duodenal biopsy samples were obtained in all dogs, 54/103 (52%) also underwent colonoscopy, and in 42 of these dogs the ileum was evaluated and biopsy samples obtained. Histopathology reports were available for review in 96/107 (89.7%) dogs. Duodenal histopathology indicated the following types of inflammation: 51 (53.1%) lymphocytic, 21 (21.9%) lymphocytic, and neutrophilic, 9 (9.4%) lymphocytic, neutrophilic, and eosinophilic, 7 (7.3%) plasmacytic, 4 (4.2%) lymphocytic, and eosinophilic, 3 (3.1%) lymphocytic, and granulomatous and 1 within normal limits. Ileal histopathology indicated the following types of inflammation: 20 (47.6%) lymphocytic, 7 (16.7%) lymphocytic and neutrophilic, 3 (7.1%) lymphocytic, neutrophilic and eosinophilic, 1 plasmacytic and eosinophilic, 1 plasmacytic and neutrophilic, and 10 (21.4%) within normal limits. Lacteal dilatation was recorded in 47/96 (49.0%), lymphangiectasia in 21/96 (21.9%), and crypt abscesses in 27/96 (28.1%) of dogs.

### 3.4 Treatment at the time of discharge or death

Mean duration of hospitalization for all dogs was 6.4 +/−5.2 days. At the time of discharge or death 27 (25.7%) were managed with diet only, 35 (33.3%) with diet and glucocorticoids, 4 (3.8%) with diet and antibiotics, 12 (11.4%) with diet, glucocorticoids and antibiotics, 20 (19.0%) with diet, glucocorticoids and a second immunosuppressive agent, 3 (2.8%) with diet, glucocorticoids, antibiotics and a second immunosuppressive agent and 4 (3.8%) with a second immunosuppressive agent only. All 4 dogs receiving only a second immunosuppressive agent received cyclosporine. The types of diet fed during hospitalization were recorded in 96 (89.7%) dogs, and were as follows: 76 (79.2%) were fed a hydrolyzed diet, 14 (14.6%) were fed a limited ingredient novel protein diet, 5 (5.2%) were fed a gastrointestinal diet and 1 dog was fed a diet classified as “other.” Twenty (18.7%) dogs had an esophagostomy feeding tube placed at the time of intestinal biopsy collection.

### 3.5 Survivors vs. non‐survivors

Twenty‐three (21.5%) dogs did not survive to hospital discharge. Of these dogs, 6 (26.1%) were euthanized because of financial limitations or failure to improve, 5 (21.7%) died of aspiration pneumonia, 3 (13.0%) died of hemorrhagic diarrhea with hypovolemia, 2 (8.7%) had a deterioration in demeanor, and in 2 (8.7%) dogs, sudden death of unknown cause was reported. A single dog died of each of the following: dyspnea, congestive heart failure, sepsis, pleural effusion, and hypernatremia with concurrent seizures.

Dogs not surviving to hospital discharge had a longer duration of clinical signs (median, 2 months; range, 0.1‐40 months; *n* = 22) vs. 1 month (range, 0.25–42 months, *n* = 83; *P* = .023) and longer duration of hospitalization (mean, 10.8 days; SD, 7.5 days; *n* = 22) vs. 5.3 days (SD, 3.7 days; *n* = 84; *P* = 0.004; Table 1). No significant differences were found between distribution of sex, BCS, presenting clinical signs, dosage of prednisolone, or use of a second immunosuppressive agent at discharge or death, type of diet fed during hospitalization, presence or absence of an esophagostomy feeding tube, or histopathologic findings between survivors and non‐survivors to hospital discharge (*P* > .06, Table 1). Six of the 23 (26.1%) non‐survivors were Pugs, and Pugs were more likely to not survive until hospital discharge when compared with all other breeds (OR, 4.93; 95% CI, 1.41–17.24; *P =* .01).

**TABLE 1:** Differences in variables between dogs with protein‐losing enteropathy caused by inflammatory enteritis, lymphangiectasia or both surviving or not surviving until hospital discharge.

Variable		Non‐survivors	*n*	Survivors	*n*	*P*‐value
Age (years)		7.8 (2.8)	23	6.9 (1.7)	84	.16
Body weight (Kg)		14.5 (10.1)	22	17.7 (12.4)	83	.3
Length of hospitalization (days)		10.8 (7.5)	22	5.3 (3.7)	84	**.004**
Duration of clinical signs (months)		2 (0.1–40)	22	1 (0.25–42)	83	**.02**
Neutrophil count (×10^9^/L)		17.5 (8.2)	22	15.3 (12.2)	71	.06
Lymphocyte count (×10^9^/L)		0.75 (0‐6.02)	20	0.81 (0‐5.48)	65	.83
Neutrophil‐to‐lymphocyte ratio		25.4 (19.7)	19	21.6 (20.7)	61	.22
Albumin (g/L)		16.3 (5.2)	23	17.7 (4.4)	83	.20
Globulin (g/L)		19.5 (4.8)	21	18.6 (5.4)	81	.25
C‐reactive protein (mg/L)		45.2 (4‐79.9)	13	18.6 (0.3‐152)	33	.19
Prednisolone dose (mg/kg/day)		1.94 (0.17)	4	2.09 (0.87)	42	.37
Dexamethasone dose (mg/kg/day)		0.29 (0.11)	12	0.38 (0.20)	8	.11
Body condition score	1/9	1 (5.0%)	20	2 (2.7%)	73	.32
	2/9	5 (25.0%)		12 (16.4%)		
	3/9	4 (20.0%)		20 (27.4%)		
	4/9	4 (20.0%)		16 (21.9%)		
	5/9	1 (5.0%)		15 (20.5%)		
	6/9	2 (10.0%)		6 (8.2%)		
	7/9	1 (5.0%)		1 (1.4%)		
	8/9	2 (10.0%)		1 (1.4%)		
Use of a second immunosuppressive agent	No 2nd agent	15 (71.4%)	21	62 (73.8%)	84	.85
	Chlorambucil	2 (9.5%)		7 (8.3%)		
	Cyclosporin	4 (19%)		14 (16.7%)		
	Azathioprine	0 (0.0%)		1 (1.2%)		
Diet type consumed	Hydrolyzed	14 (82.3%)	17	62 (78.5%)	79	.12
	Gastrointestinal	1 (5.9%)		4 (5.1%)		
	Limited ingredient novel protein	1 (5.9%)		13 (16.4%)		
	Other	1 (5.9%)		0 (0.0%)		
Sex	Male intact	7 (30.4%)	23	8 (9.5%)	84	.14
	Male neutered	12 (52.1%)		39 (46.4%)		
	Female intact	1 (4.3%)		4 (4.8%)		
	Female neutered	3 (13.0%)		33 (39.3%)		
Treated with steroids	Yes	17 (81.0%)	21	53 (63.1%)	84	.09
	No	4 (19.0%)		31 (36.9%)		
Esophagostomy tube placed	Yes	6 (26.1%)	23	14 (16.7%)	84	.31
	No	17 (73.9%)		70 (83.3%)		

*Note*: Significant *P*‐values are listed in bold. Continuous values reported as mean (+/−SD) if normally, and median (range) if not normally distributed. Categorical values represented as frequency (percentage).

### 3.6 Change in serum CRP

**FIGURE 1:**
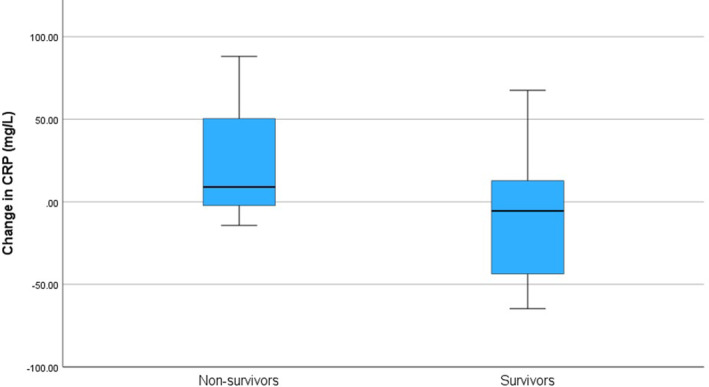
The difference in change of serum C‐reactive protein (CRP) concentration from admission to 1–3 days of in‐hospital treatment between dogs with protein‐losing enteropathy surviving or not surviving until hospital discharge. A significant difference was found in the change in CRP from day 0 to 1‐3 days after treatment in‐hospital between survivors and non‐survivors: −11 (+/−40.8) mg/dL vs. +28 (+/−39.1) mg/dL (*P* = .02).

Serum CRP concentrations were measured more than once during hospitalization in 26 dogs (Table 2). The change in CRP between day 0 and 1–3 was different between dogs surviving or not surviving to hospital discharge (mean, − 11 mg/dL; SD, 40.8 vs. mean, +28; SD, 39.1; *P* = .02; Figure 1). Change in CRP concentration between days 0 and 4–7 after treatment in‐hospital was not significantly different between survivors and non‐survivors (*P* = .07).

**TABLE 2:** Serum C‐reactive protein concentrations during hospitalization in dogs with protein‐losing enteropathy caused by inflammatory enteritis, lymphangiectasia or both with measurements at day 0 or day 1–3 and day 4–7 after initiating treatment in hospital between dogs surviving or not non‐surviving to hospital discharge.

	Non‐survivors	*n*	Survivors	*n*	*P*‐value
Day 0 CRP (mg/dL)	17.5 (8.2)	11	15.3 (12.2)	15	.36
Day 1–3 CRP (mg/dL)	71.6 (45.5)	8	43.4 (28.5)	8	.09
Day 4–7 CRP (mg/dL)	34.1 (26)	3	25.3 (14.7)	7	.41

*Note*: Values reported as mean (+/−SD).

### 3.7 Pugs survival to discharge

Twelve Pugs were included and 6/12 (50%) did not survive until hospital discharge. Reported causes of mortality in Pugs were aspiration pneumonia in 5/6 (83.3%) and hypernatremia and associated seizures in 1. Pugs that did not survive until hospital discharge were older (mean, 9.4 years; SD, 1.5 vs. mean, 5.8 years; SD, 2.2; *P* = .004), had a longer duration of hospitalization (mean, 8.2 days; SD, 3.7 vs. mean, 3.5 days; SD, 1.0; *P* = .01), higher neutrophil counts (mean, 23.5 × 10^9^/L; SD, 9.4 vs. mean, 14.3 × 10^9^/L; SD, 5.2; *P* = 0.03), higher NLR (mean, 24.3; SD, 10.6 vs. mean, 15.2; SD, 6.2; *P* = .05), and higher CRP concentrations (mean, 50.5 mg/L; SD, 22.6 vs. mean, 17.7 mg/L; SD, 9.3; *P* = .03) at the time of admission compared with Pugs that did survive until hospital discharge (Table 3). No significant differences were found in body weight, duration of signs, lymphocyte count, or serum albumin or serum globulin concentrations between surviving and non‐surviving Pugs (*P* > .16; Table 3). Only 1 Pug received a second immunosuppressive agent; this dog received cyclosporine and survived until hospital discharge.

**TABLE 3:** Differences in variables between Pugs with protein‐losing enteropathy caused by inflammatory enteritis, lymphangiectasia or both surviving or not surviving until hospital discharge.

	Non‐survivors	*n*	Survivors	*n*	*P*‐value
Age (years)	9.4 (1.5)	6	5.8 (2.2)	6	**.004**
Body weight (kg)	8.7 (1.5)	6	9.7 (2.3)	6	.2
Length of hospitalization (days)	8.2 (3.7)	6	3.5 (1.0)	6	**.01**
Duration of clinical signs (months)	1.5 (0.3–42.0)	6	1.0 (0.5–2.0)	6	.16
Neutrophil count (×10^9^/L)	23.5 (9.4)	6	14.3 (5.2)	6	**.03**
Lymphocyte count (×10^9^/L)	1.09 (0.50)	6	1.02 (0.32)	6	.78
Neutrophil‐to‐lymphocyte ratio	24.3 (10.6)	6	15.2 (6.2)	6	**.05**
Albumin (g/L)	12.5 (4.0)	6	13.9 (4.6)	6	.3
Globulin (g/L)	20.1 (4.4)	6	17.9 (5.6)	6	.23
C‐reactive protein (mg/L)	50.5 (22.6)	5	17.7 (9.3)	6	**.03**

*Note*: Significant *P*‐values are listed in bold. Values reported as mean (+/−SD) if normally, and median (range) if not normally distributed.

## 4 DISCUSSION

Despite studies evaluating the associated mortality and predictors of mortality in dogs with PLE, information regarding in‐hospital mortality and associated factors is lacking. Our aims were to report the in‐hospital mortality rate of dogs with PLE, causes for mortality and any associated risk factors.

In our study population, 23/107 (21.5%) dogs did not survive until hospital discharge. These results suggest that PLE in dogs is associated with substantial in‐hospital mortality. However, our study population may be biased towards a higher mortality for several reasons. A referral population was used in our study, which may have selected for more severely affected dogs. To exclude dogs with intestinal neoplasia, only dogs with a histopathologic diagnosis were included. Dogs with less severe disease may not undergo biopsy, thus selecting against dogs that may have more favorable in‐hospital survival. Increased mortality may be associated with general anesthesia and obtaining intestinal biopsy specimens. This mortality could occur through increased risk of aspiration pneumonia,^16^, ^17^, ^18^ or through exacerbation of an underlying hypercoagulable or immunocompromised state, both of which have been identified in dogs with PLE.^11^, ^13^ Obtaining endoscopic biopsy specimens generally is considered safe, but complication rates are not well described in dogs. In a previous study investigating complications associated with colonoscopy in dogs, only 0.85% were reported to have major complications.^19^ When complications do occur, they can be associated with a favorable prognosis.^20^ The contribution of these procedures to mortality in our study cannot be fully determined, and additional studies comparing the mortality of dogs diagnosed with PLE that did or did not undergo biopsy should be performed.

The most commonly recorded cause of mortality in our study population was a decision to euthanize for financial reasons or failure to improve with treatment. These 2 causes of mortality were grouped together, because retrospectively it was difficult to differentiate them. Some of these dogs may have survived until discharge should treatment have been continued. Failure to improve or decision to euthanize also could be associated with deterioration or a complication not recorded in the hospital records. The second most common cause of hospital mortality was aspiration pneumonia, and all affected dogs were Pugs. The diagnosis of aspiration pneumonia in all of these dogs was based on compatible imaging findings; 4/5 using point‐of‐care ultrasonography and 1/5 using thoracic radiography. None of these dogs underwent airway sampling or necropsy examination to confirm the diagnosis of aspiration pneumonia. All dogs diagnosed with aspiration pneumonia using point‐of‐care ultrasonography had a shred sign reported. The specificity of a shred sign for the diagnosis of bacterial pneumonia previously has been reported as 92%.^21^ However, given the retrospective nature of our study, images cannot be reviewed and a standardized approach and definition of findings using thoracic ultrasonography cannot be confirmed.

Common risk factors for aspiration pneumonia include gastrointestinal disease, general anesthesia and brachycephalic conformation.^16^, ^17^, ^18^, ^22^ Interestingly, this complication was only associated with Pugs, despite there being 14 other brachycephalic dogs in the study population. Other possibilities are that aspiration pneumonia was over diagnosed, and that respiratory deterioration could have been associated with pulmonary thromboembolism, as has been previously suspected in dogs with PLE.^12^ Alternatively, point‐of‐care ultrasonography may have accurately identified subclinical aspiration pneumonia, but it may not have been the true reason for deterioration. All of the dogs not surviving as a result of aspiration pneumonia were receiving corticosteroids during hospitalization, which may have exacerbated subclinical aspiration pneumonia or contributed to worsening of their condition. The dosages of dexamethasone ranged from 0.1 to 0.3 mg/kg/day, and 1 dog was receiving prednisolone PO at a dosage of 1.9 mg/kg/day. These findings emphasize the importance of decreasing the risk of aspiration in Pugs undergoing investigations for PLE, and judicious use of immunosuppressants in this population.

A number of other variables were different between survivors and non‐survivors, including duration of hospitalization, duration of clinical signs, and change in CRP after treatment. Increased duration of hospitalization in dogs not surviving to discharge likely reflects prolonged hospitalization associated with increased cost, morbidity, and lack of clinical improvement. Dogs that did not survive until hospital discharge had a longer duration of clinical signs before admission, which may have contributed to malnutrition, a factor that has been shown to be associated with decreased long‐term survival in dogs with PLE.^6^ Alternatively, dogs that are refractory to treatment may have a more protracted history. There was almost complete overlap in the range of duration of signs between survivors and non‐survivors, suggesting it would not be an accurate variable to estimate prognosis, but it does emphasize the importance of punctual investigation and treatment in dogs with PLE.

No difference was found between the type of diet consumed, corticosteroid dosage or the use of a second immunosuppressive agent when comparing survivors and non‐survivors. Although not statistically significant, a higher percentage of non‐survivors received glucocorticoids compared with survivors (81.0% vs. 63.1%). The use of diet alone in the treatment of PLE is associated with a more favorable long‐term outcome than observed in dogs managed with glucocorticoids.^23^ The majority of dogs not surviving until hospital discharge were treated with glucocorticoids, which may reflect the severity of their disease or an escalation of treatment after deterioration or lack of improvement in the hospital. The absence of significance suggests that the use of diet instead of glucocorticoids is more important when considering longer term outcome. Placement of an esophagostomy feeding tube was not associated with improved survival, despite a previous study that reported an association with assisted enteral nutrition and positive outcome at 6 months.^24^ This finding also emphasizes that placement of an esophagostomy feeding tube does not increase in‐hospital mortality and should be considered when appropriate. Lastly, this finding supports previous studies documenting that esophagostomy feeding tube placement typically is not associated with life‐threatening complications.^25^

CRP has been used as an indicator of inflammation,^26^, ^27^ and monitoring changes in CRP has been associated with treatment response when managing dogs with pneumonia.^28^, ^29^ Assessing the change in CRP during hospitalization was therefore of interest in dogs treated for PLE. Routine measurement of CRP at our institution began in 2019 and therefore most dogs in our study had no CRP measurements. Only 26 dogs had repeat CRP measurements during hospitalization, and selection bias likely occurred towards dogs not improving or deteriorating, because such a situation may lead clinicians to repeat serum biochemistry including CRP. C‐reactive protein concentration at the time of admission was not associated with mortality during hospitalization, but such an association was found with a change in CRP between admission and 1–3 days after treatment. Previous studies in dogs have not identified an association between CRP and long‐term outcome in dogs with PLE,^3^ but CRP normalized in all of the evaluated dogs. The association with a change in CRP and outcome in our study may reflect rapid improvement in intestinal inflammation in dogs that responded and survived until discharge. Dogs in which CRP did not decrease, not only may have shown a lack of improvement in their intestinal inflammation, but also may have developed inflammation or infectious co‐morbidities, such as aspiration pneumonia. A change in CRP from admission to 4–7 days after treatment was not predictive of survival until discharge. Reasons for this finding may include a type II error, because only 3 dogs not surviving until discharge were included in this group. C‐reactive protein concentration may also only be applicable to certain causes of mortality. The reported causes of death for these 3 dogs were failure to improve for 2, and sudden death for 1 dog. Future studies are needed to explore the utility of CRP in predicting specific outcomes and causes of death in dogs with PLE.

Pugs were overrepresented in our study population and experienced increased mortality, compared with non‐Pug breeds. Reasons for this difference may include a higher risk of aspiration pneumonia, but previous reports have not identified a higher risk in Pugs compared with other brachycephalic breeds.^22^ Five of 6 Pugs were reported to have died of aspiration pneumonia, and they were the only breed reported to do so. As previously discussed, this finding may represent a bias or over diagnosis of aspiration pneumonia in this breed. Non‐survivors however were shown to have an increase in neutrophil counts and CRP at the time of admission, which was in contrast to findings when analyzing all breeds. Therefore, if aspiration pneumonia is the cause of mortality in these dogs, it may suggest that undiagnosed aspiration was already present at the time of admission. This morbidity then may be exacerbated by general anesthesia, and worsened by corticosteroid treatment, which all Pugs were receiving at the time of death. Other reasons for increased mortality may be associated with the underlying cause of PLE. It has been reported that some breed‐specific causes of PLE may not require immunosuppressive treatment.^30^ Overall, our findings emphasize the importance of investigating the cause of increased CRP in Pugs presenting with PLE, and additional studies should evaluate the utility of thoracic imaging before treatment and the risks associated with immunosuppression in such dogs.

Limitations of our study include its retrospective design and reliance on hospital records, which were not always complete. Also, the histopathologic diagnosis sometimes depended on what had been recorded in the discharge report as opposed to the written laboratory report. The cause of death was determined from the hospital records, and none of these dogs underwent necropsy examination. Thus, the suspected cause of death could not be confirmed. Imaging findings to determine the diagnosis of aspiration pneumonia largely were based on point‐of‐care ultrasonography, and these images were not available for retrospective review. The retrospective nature of our study also meant we were unable to determine the effect of caloric intake and dietary fat content during hospitalization on outcome. Although histopathologic diagnosis was required, in most cases the ileum and jejunum were not biopsied and therefore the presence of neoplasia in these sections of the intestine cannot be excluded. Also, given the retrospective nature of our study, we were unable to reliably assess other predictors of negative outcome in dogs with PLE, including CCECAI, vitamin D concentrations, small intestinal dilatation, epaxial muscle loss, and hair coat quality.^3^, ^4^, ^5^, ^6^, ^7^, ^8^, ^9^, ^10^ Finally, when performing statistical analysis, the decision was made not to correct *P*‐values for multiple comparisons, because of sample size, and therefore concern for type II error. In addition, given the sample size, we were unable to perform logistic regression, because the final number of dogs in the outcome group of not surviving until hospital discharge that had complete data and could be included in the models was only 15. Therefore, based on this number, only 1.5 variables could have been included in multivariable analysis.^31^, ^32^ A multicenter study allowing for a larger data set is needed to corroborate our findings and determine which variables are independent of each another.

In conclusion, we demonstrated that the in‐hospital mortality of dogs with PLE caused by inflammatory enteritis, lymphangiectasia or both presented to a referral hospital was 21%. The most common causes of mortality were financial limitations, failure to improve, and aspiration pneumonia. Finally, variables associated with mortality during hospitalization included longer duration of hospitalization, longer duration of clinical signs and an increase in CRP 1–3 days after treatment. Higher mortality was identified in Pugs and was most commonly attributed to aspiration pneumonia.

## CONFLICT OF INTEREST DECLARATION

Authors declare no conflict of interest.

## OFF‐LABEL ANTIMICROBIAL DECLARATION

Authors declare no off‐label use of antimicrobials.

## INSTITUTIONAL ANIMAL CARE AND USE COMMITTEE (IACUC) OR OTHER APPROVAL DECLARATION

Ethical approval was not required as determined by the Clinical Research Ethical Review Board at the Royal Veterinary College.

## HUMAN ETHICS APPROVAL DECLARATION

Authors declare human ethics approval was not needed for this study.
